# Aroma Perception of Limonene, Linalool and α-Terpineol Combinations in Pinot Gris Wine

**DOI:** 10.3390/foods12122389

**Published:** 2023-06-16

**Authors:** Mildred Melina Chigo-Hernandez, Elizabeth Tomasino

**Affiliations:** Department of Food Science & Technology, Oregon State University, Corvallis, OR 97331, USA

**Keywords:** pinot gris, monoterpenes, limonene, triangle test, check-all-that-apply, aroma interaction

## Abstract

Aromatic white wines contain monoterpenes that can alter aroma qualities based on their concentration and enantiomeric ratios. Limonene has been identified as a monoterpene that is used to differentiate monovarietal white wines. The aim of this study was to evaluate the influence of limonene on aroma perception at different enantiomeric ratios. Its interaction with linalool and α-terpineol compounds was also investigated. Eighteen model wines were created with different ratios and/or concentrations of limonene and diverse concentrations of linalool and α-terpineol. Triangle tests, check-all-that-apply (CATA) and descriptive analysis were used to evaluate the aroma of the wines. Results show that different limonene ratios had no influence on wine aroma. Descriptive analysis showed that the addition of only limonene influenced citrus characteristics depending on the concentration. Linalool addition did not alter aroma quality when the limonene was at low concentrations, but it did change aroma perception at high limonene levels. α-Terpineol only altered the aroma of the wine at medium and high concentrations. At high concentrations, linalool and α-terpineol presented tropical aromas with some floral notes, irrespective of limonene levels. Depending on the desired aroma qualities of the wine, altering the monoterpene content resulted in very different aromatic wines.

## 1. Introduction

Monoterpenes are volatile compounds found in grapes and are fundamental aroma compounds for varietal wines such as Muscat, Riesling, Chardonnay, Pinot gris, and Gewürztraminer [1,2,3]. These compounds can have one or more pairs of enantiomers; each pair of enantiomers can have a unique aroma that contributes to perception through orthonasal olfaction and flavor via retronasal olfaction [3,4,5]. Monoterpene mixtures can also be attributed to aroma qualities not associated with the individual compounds [6,7].

In wines, the effect of combining such a large variety of aroma compounds makes differentiating the causes of specific aromas a complex process. The high degree of complexity has kept much of the research on aroma causes in wine focused on individual aroma compounds rather than their combined perceptions [8]. The influence of individual monoterpenes in white wines has been extensively studied, though a few have investigated mixtures of monoterpenes’ impacts on aromas [9,10].

Chemical composition and aroma perception have also been major research focuses for their potential impacts using odor activity values (OAV) or the aroma thresholds of compounds [11]. OAVs also utilize the threshold of the target compound, with concentrations above the threshold considered to be very impactful to aroma perception over those found at concentrations below their threshold. OAVs and compound perception thresholds are problematic for determining the cause of aromas in wine, as are that the thresholds are typically determined in air, water or a simple ethanol solution, not in wine. Research has shown that the thresholds of compounds in wine are very different from those determined in simple solutions [11,12,13]. These methods also do not account for how perception changes when compounds are found in mixtures and, therefore, are not appropriate for determining the cause of aromas in complex mixtures.

Pinot gris is an aromatic wine originating from France and typically has honey and apricot aromas [3,14]. Many monoterpenes have been identified in Pinot gris wines, including α-terpineol, citronellal, linalool, geraniol, and limonene [3,7,15,16]. Of these compounds, linalool and α-terpineol have been found to be influential to the aroma of other white wines [6]. In Pinot Gris wines, linalool and α-terpineol were found to have similar ratios of enantiomeric pairs, but a range of enantiomeric ratios for limonene, from 33(−):67(+) to 79(−):21(+) have been found [7]. (−)-Limonene is known to have a threshold of 500 μg/L and is described as a turpentine-like aroma. R-(+)-limonene has a threshold of 200 μg/L and is described as having a fresh, orange-like aroma [17,18,19]. Therefore the differences in enantiomer ratios in Pinot gris has the potential to alter aroma perception.

The objectives of this research were: (1) to evaluate the sensory perception of four ratios of limonene to assess its aroma quality differences and (2) to obtain the aroma quality of limonene when in mixtures with other monoterpenes (linalool and α-terpineol). This work focuses on expanding our understanding of how monoterpenes influence the aroma perception of white wine. By understanding the causes of specific aroma qualities, it will be possible to determine how viticultural and winemaking processes may alter those qualities in future research.

## 2. Materials and Methods

### 2.1. Chemicals

The following chemical standards were used in the aroma base and were obtained from Sigma-Aldrich Co. (St. Louis, MO, USA): ethanol (≥99%), 2,3-butanedione (97%), hexan-1-ol (98%), methionol (≥98%), butanoic acid (≥99%), decanoic acid (≥98%), 2-methylpropanoic acid (99%), 2-methylbutanoic acid (98%), 3-methylbutanoic acid (99%), 2-methylpropyl ethanoate (≥97%), ethyl phenylacetate (≥99%), ethyl 3-methylbutanoate (98%), ethyl 2-methylpropanoate ((≥98%), ethyl 2-methylbutyrate (99%), ethyl butanoate (≥98%), ethyl decanoate (≥99%), ethyl hexanoate (≥99%), ethyl octanoate (≥98%), hexanoic acid (99%), octanoic acid (≥99%), 2-methylpropan-1-ol (≥99%), ethyl acetate (≥99%) and isoamyl acetate (≥99%). Acetic acid (≥99%) was obtained from VWR International (Radnor, PA, USA). The terpenes compound added to the model wine for the treatments were: (S)-(−)-limonene (96%), (R)-(+)-limonene (97%), linalool (≥97%) and α-terpineol, which were purchased from Sigma-Aldrich Co. (St. Louis, MO, USA).

### 2.2. Wine Base

Wine aroma compounds were removed using two methods. Aroma compounds were removed from the wine as described by Tomasino et al. [10]. LiChrolut EN resin was added to the wine at a rate of 1.5 g L^−1^. Wines with resin were stirred for 24 h before filtering out the resin. Dearomatized wines were checked using GCMS prior to use to ensure aroma compounds were removed. Dearomatized wines were stored in stainless ball lock kegs (AMCYL, Wyoming, MN, USA) sparged with nitrogen at 4 °C for later use.

A week prior to sensory analysis, the aroma base (Supplementary Table S1) was added to the dearomatized wine from stock standards for each compound. This model wine was then bottled in 750 mL glass wine bottles with screw caps (Stelvin Amcor, Zurich, Switzerland) and stored at 4 °C until the sensory panel.

### 2.3. Standards and Wine Models

Stock standard solutions of all chemicals used in the aroma base and wine models were prepared as described by Chigo-Hernandez et al. [6]. Concentrations of the terpenes in each treatment were chosen according to concentrations found in the literature and measured in wines (Table 1) [10].

### 2.4. Sensory Analysis

All sensory analysis was approved by the Oregon State University Internal Review Board (#8606). Panelist inclusion criteria, room parameters and software are the same as described in Chigo-Hernandez et al. [6].

### 2.5. Triangle Test Procedures

For triangle tests, 40 women, 18 men and 1 non-binary person, all above 21 years old, for a total of 59 participants, were recruited from Oregon State University and the Corvallis, Oregon area. The triangle tests evaluated the 4 wines with different Limonene enantiomer ratios (Table 1). This was performed to determine if the ratio of limonene enantiomers elucidated changed aroma sensory perception. Triangle tests were conducted between November 10 and 17 November, 2021. Six triangle tests (all possible combinations) were presented in one sensory session.

Black INAO wine glasses (Lehmann glass, Kiyasa Group, New York, NY, USA) labeled with three-digit random codes and covered with plastic lids (Clark Associates, Inc., Lancaster, PA, USA) were used to serve the wines. Wines were poured in 20 mL aliquots 30 min before each sensory test. Panelists smelled the sample and indicated the wine that was most different. A one-minute break between each test was required to avoid any carryover effects and fatigue.

### 2.6. CATA

The ratio of the limonene found most in research was used based on triangle test results (Section 3.1) [7]. The original concentrations chosen for linalool and α-terpineol (Supplementary Table S2) did not result in any statistical differences using CATA (CATA1), and therefore, increased concentrations were used in a second CATA panel (CATA2). These concentrations are still concentrations measured in Pinot gris wines [7]. The final treatments evaluated for CATA were models 5 to 18, where 18 was the aroma base with no monoterpenes added. The CATA included 24 sensory descriptors with two “other” options to avoid any “dumping effect” [20]. CATA analysis occurred in the same room as the triangle test at OSU 6 and 8 of December 2021 for CATA1 and on 26 and 28 January 2022 for CATA2. For CATA1, 25 wine consumers (18 women, 6 men and one non-Binary person, all above 21) participate in two sessions (1-h sessions). Panelists were instructed to smell the sample and select all the descriptors that were perceived in the sample. A 30-s break between each test was required to avoid any carryover effects and fatigue. For CATA2, 27 wine consumers (20 women, 6 men and one non-binary person, all above 21) followed the same instructions as the CATA1 panel.

### 2.7. Descriptive Analysis for Aroma Intensity

From the CATA2 results, 13 aroma descriptors were selected for further descriptive analysis using line scales. Attributes with their training standards and images used in training can be found in Table 2. Twenty-three wine consumers (20 female, 7 males, all above 21 were trained on recognizing 13 different aroma standards. Participants were trained through multiple choice odor and image recognition training as described by Tomasino et al. [10].

### 2.8. Statistical Analysis

Triangle tests, CATA and PCA results were calculated as described in Chigo-Hernandez et al. [6].

## 3. Results and Discussion

### 3.1. Triangle Tests

Limonene has been found in Pinot gris at different ratios; this is an important aspect to investigate since the variation in enantiomers percentages can lead to a diversity of aroma perceptions when in combination with other mixtures [6,7]. Four enantiomer ratios (33:67, 55:45, 67:33 and 79:21) were evaluated in a dearomatized white wine with the same added aroma base. Triangle tests (Table 3) showed no significant differences providing evidence that the different limonene enantiomer ratios did not have the capacity to alter the aroma perception in a Pinot gris wine. Sensory differences by limonene enantiomer ratios have been found but at much higher concentrations than those used in this study [21]. The concentrations of limonene used in this study were below the limit of reported perception thresholds [17,18,19,22] but were chosen as they were realistic to the amounts measured in Pinot gris [7]. These lower concentrations were also chosen as it is known that the addition of other aroma compounds, such as those in the aroma base, could result in a change in the limonene threshold [23,24]. Rapp [9] showed the threshold of linalool decreased when linalool was in combination with other monoterpenes, but this is unknown for limonene. Furthermore, limonene enantiomers have different threshold values; for instance, R-(+)-limonene has a 200 µg/L threshold, and S-(−)-limonene has a 500 µg/L [17,18,19,25]. The lack of differentiation in triangle test results may be because the threshold of one of the enantiomers was reached but not the other or that the threshold was not altered despite being in combination with other aroma compounds. Additionally, the concentrations used did not reach the differential threshold for these compounds or just-noticeable a difference, which is the minimum physical change required to sense the change half of the time [26]. Since no differences were found, the model that contained the most enantiomer ratio found in the literature was used for the further sensory panel, 55:45, in favor of the (−) enantiomer.

### 3.2. CATA

CATA was specifically used to determine aromas for further descriptive analysis investigating the intensities of specific aromas. The original compound concentrations used for CATA1 showed no statistical significance. The fact that the concentrations used in CATA1 showed no sensory differences suggests we did not reach the differential or perception thresholds for these compounds, as stated above for triangle tests [26].

**Figure 1:**
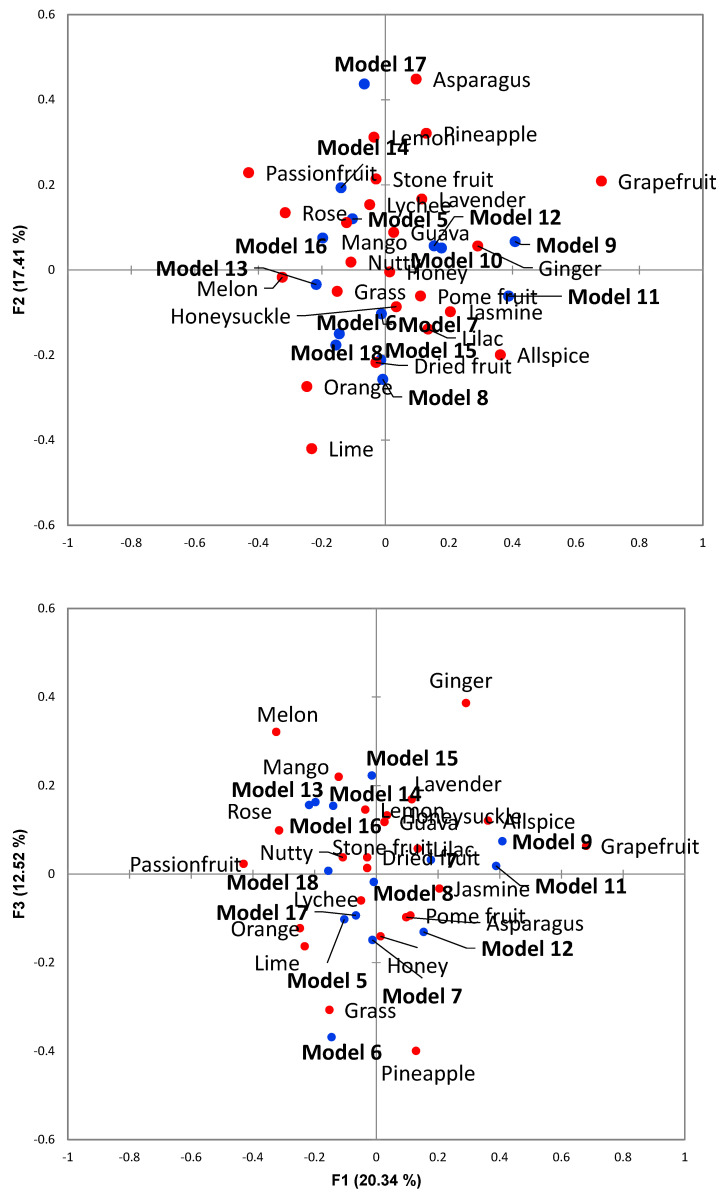
Correspondence analysis of descriptors used from CATA 2 analysis evaluating wines with different combinations of terpenes.

It was not until the concentrations were increased in CATA 2 that aroma perception differences were achieved. The increased concentrations are still within those measured in Pinot gris wines [7]. The results for CATA2 had 16 attributes used more than 15% of the total terms chosen. It was still necessary to reduce the number of terms for further descriptive analysis, as large numbers of terms can result in fatigue and other issues. Cochran’s Q-test (Supplementary Table 3) and associations shown in correspondence analysis (Figure 1) were used to reduce terms.

The first three variates of correspondence analysis explained 50.28% of the total variance (Figure 1). Based on the Scree plot for the analysis, additional dimensions beyond the first three did not add greatly to the total variance. Certain descriptors can be clearly associated with the different models (Figure 1); Model 18 (Aroma base) was described with citrus notes, and Models 14, 16 and 13 were similarly located and described with melon, mango and rose aromas. Models 15 and 18 were similar and described by dried fruit aroma. Known similar terms were combined in accordance with previous sensory work [10,20]. e.g., pineapple and mango were combined into tropical fruit. In total, 13 terms were used for further descriptive analysis (Table 2).

### 3.3. Descriptive Analysis

**Figure 2:**
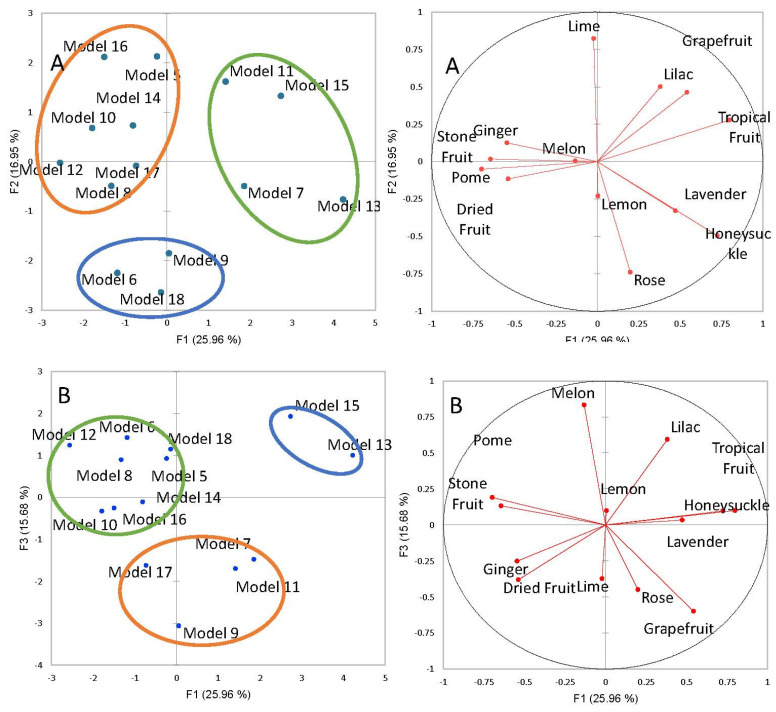
Separation of wine models by monoterpene profiles using PCA. Circles with the model wine results represent the clusters obtained by K-means clustering. (**A**) displays resuls for F1 and F2 and (**B**) provides results for F1 and F3.

Differences were found between the model wines; the first two variates of discriminant analysis explained 43% of the total variance. A third dimension was included to increase the total variance to 58.7%. Based on the Scree plot for the analysis, additional dimensions did not add greatly to the total variance after the 3rd dimension. The wine models were classified into three clusters, using K-means clustering (Figure 2), although depending on which F1, F2 or F3 axis are viewed, which wines are in which cluster does vary. The greatest variance (F1 vs. F2) results in one cluster (blue) with models 6, 9 and 18, another cluster (green) with models 7, 11, 13 and 15, and the third cluster (orange) contain models 5, 8, 10, 12, 14, 16 and 17.

Along F1 and F2, the blue cluster sits between multiple vectors, suggesting some floral, dried fruit and lemon aromas. By incorporating F3, it appears that model 9 is characterized by a lime aroma and models 6 and 18 by a melon aroma. While models 7, 11, 13 and 15 are in one cluster (green), models 7 and 13 are characterized more by lavender and honeysuckle aromas and models 11 and 15 by grapefruit, tropical fruit and some lilac aromas. By incorporating F3, it appears that models 15 and 13 are more associated with floral and tropical fruit aromas and models 11 and 7 also have rose and lime characteristics. The orange cluster, with the greatest number of models, is characterized by ginger, stone fruit, pome, and dried fruit aromas. These same descriptors are associated with the models when including F3.

To compare the different wine models, it is important to take the results across the three dimensions into consideration. The aroma base model (model 18) has clear lemon and melon notes, and when both low and high concentrations of limonene (models 5 and 9) are present, citrus aromas (lime and lemon) are dominant. This suggests that the addition of limonene does alter the aroma away from melon to more dominant citrus aromas. This was anticipated as limonene is found in the peel of citrus fruits [27], and it has been described when alone as having citrus and herbal aromas [28]. However, it is unclear if high limonene concentrations impart a significant influence on the overall aroma, as model 9 was in the same cluster as model 18. Our results suggest that more limonene increases the already present citrus aromas.

By comparing model 7 and model 17, it can be seen that the addition of limonene to the same concentration of α-terpineol shows a shift from pome, lime, and dried fruit aromas to more floral and grapefruit aromas. An increase in the concentration of limonene to high levels when the concentration of α-terpineol is reduced does not result in a change in aroma since models 7 and 11 are in the same cluster across the three dimensions. It is when α-terpineol is increased to high concentrations (model 14) does the aroma alter to ginger, lime and dried fruit. These are the aroma descriptors for α-terpineol in F3 (model 17), indicating that high limonene concentration increases these aromas. α-Terpineol on its own is associated with lilac aromas [23], which was perceived in model 11. α-Terpineol has also shown lemon and pome aromas when in a Gewürztraminer aroma wine base model, but it is unclear if this is different from the aroma base used since this was not evaluated in this study [6].

The wine model with linalool at medium concentrations presented ginger aromas. This is interesting since linalool, in combination with geraniol, are the main aroma compounds associated with ginger [23]. The addition of limonene to medium levels of linalool (models 16 and 6) also showed a shift in aroma quality. Limonene appears to mask the contribution of linalool as model 6 is clustered with model 9, only described as lemon, and model 18, the aroma base wine. When linalool is at high concentrations and in combination with limonene, linalool starts to contribute its floral aromas, found in model 13 across three dimensions. Linalool is a compound that is frequently associated with floral aromas [28]. It has also shown pome and lime aromas when in combination with other monoterpenes but only at higher concentrations than those tested in this study [6].

When all three compounds are found together, models 8, 12 and 15, several things can be noted. When both α-terpineol and linalool are in combination with limonene but at low and medium concentrations, the aromas are similar as they are found in the same cluster. When all compounds are at high concentrations, tropical fruit, lilac and honeysuckle aromas are noted. This is interesting as these compounds are not traditionally thought to cause tropical fruit aromas. As stated previously, linalool and α-terpineol are associated with floral aromas, and they are most likely contributing to the lilac and honeysuckle aromas noted in this study [29]. Although, in a previous study, a tropical fruit aroma was noted when α-terpineol and linalool were present at a higher concentration than in this study and when in combination with rose oxide without any detection of floral notes [6]. This suggests higher concentrations of these compounds could lead to tropical fruit masking other aromas, such as the citrus notes found to be caused by limonene.

## 4. Conclusions

This work has shown that the different enatiomer ratios of limonene do not alter the aroma of Pinot gris wine at concentrations measured in these wines. Additionally, limonene only contributes to the aroma perception of Pinot gris wines when other monoterpenes are found at low or medium concentrations. Once linalool and α-terpineol are found at higher concentrations, they mask any aromas that are caused by linalool. The information in this work helps to provide an understanding of how aroma chemical data can be interpreted, as well as providing additional support that high concentrations of linalool and α-terpineol in combination are a cause of tropical fruit aromas in white wines. Understanding the causes of specific aroma qualities will allow winemakers to choose the process to achieve their desired qualities for their wine process.

## Figures and Tables

**Table 1:** Concentrations (μg/L) of terpenes in each wine model.

Wines	(*S*)-(−)-Limonene	(*R*)-(+)-Limonene	Linalool	α-Terpineol
Model 1 ^a^	1.32	2.68		
Model 2 ^b^	2.2	1.8		
Model 3 ^c^	2.68	1.32		
Model 4 ^d^	3.16	0.84		
Model 5 ^b^	1.1	0.9		
Model 6 ^b^	1.1	0.9	25	
Model 7 ^b^	1.1	0.9		30
Model 8 ^b,e^	1.1	0.9	25	30
Model 9 ^b^	4.95	4.05		
Model 10 ^b^	4.95	4.05	13	
Model 11 ^b^	4.95	4.05		15
Model 12 ^b,f^	4.95	4.05	13	15
Model 13 ^b^	4.95	4.05	45	
Model 14 ^b^	4.95	4.05		62
Model 15 ^b,g^	4.95	4.05	45	62
Model 16			25	
Model 17				30
Model 18				

The wine models that had limonene had 4 ratios: ^a^ 33:67, ^b^ 55:45, ^c^ 67:33 and ^d^ 79:21. ^e^ Medium concentration of linalool and α-terpineol, ^f^ low concentration of linalool and α-terpineol, ^g^ high concentration of linalool and α-terpineol.

**Table 2:** Standards and images used for training of descriptive analysis panel.

Attribute	Amount Per Glass	Components	Image
Dried Fruit ^a^	1 tsp *	Golden raisins mixed with DI water	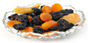
Honey Suckle	2 drops in cotton	Honeysuckle essential oil ^b^	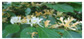
Stone Fruit	1 tsp of each	White peach ^c^ and apricot puree ^c^	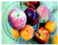
Tropical Fruit	1 tsp of each	Mango ^c^ and passion fruit puree ^c^	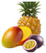
Pome	1 tsp of each	Green apple ^c^ and pear puree ^c^	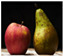
Rose	4 drops	Rosewater concentrate ^d^	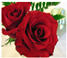
Melon	1 tsp and 1 drop of each	Honeydew puree ^e^ made and melon essential oil ^f^	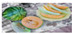
Ginger	1 tsp	Ground ginger ^g^	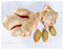
Lemon	1 tsp	Lemon pure made ^h^ and lemon essential oil ^b^	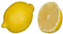
Grapefruit	1 tsp	Grapefruit pure made ^h^	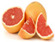
Lime	1 tsp	Lime pure made ^h^	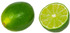
Lilac	1 drop in cotton	Lilac essential oil ^i^	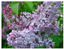
Lavender	1 drop in cotton	Lavender essential oil ^i^	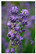

tsp * = teaspoon, ^a^ Dried fruit was pureed with the addition of distilled water one day before each sensory session and kept at refrigeration temperatures until analysis. The components were sourced from: Rainbow Abby 2013 (Guangzhou, China) ^b^, the perfect purée of Napa Valley (Napa, CA, USA) ^c^, Nielsen-Massey (Waukegan, IL, USA) ^d^, Silver Cloud Flavors (Belcamp, MD, USA) ^f^, Private selection (Cincinnati, OH, USA) ^g^, Barnhouse blue (San Clemente, CA, USA) ^i^. Puree ^e^ was made by removing the seeds and ridding of them. Purees ^h^ were made using the whole fruit. All purees were kept frozen at −23 °C and defrosted one day before each training and kept in refrigeration at 4 °C between sessions.

**Table 3:** Triangle test results for the models with different limonene ratios, performed by *z*-test (*n* = 59, for all tests).

Comparison	Number of Participants	Number of Correct Responses	*p*-Value
Model 1 vs. Model 2	59	19	>0.5
Model 1 vs. Model 3	59	14	>0.6
Model 1 vs. Model 4	59	17	>0.7
Model 2 vs. Model 3	59	24	0.14
Model 2 vs. Model 4	59	19	>0.9
Model 3 vs. Model 4	59	17	>0.10

## Data Availability

The data presented in this study are available on request from the corresponding author.

## Supplementary Materials

The following supporting information can be downloaded at: https://www.mdpi.com/article/10.3390/foods12122389/s1, Table S1—Final concentration and CAS# of aroma compounds used in the wine base; Table S2—Concentration (μg/L) of terpenes used for CATA1 sensory panel; Table S3—Multiple pairwise comparisons using the critical difference (Sheskin) procedure.
