# Supplementary material for: Aroma Perception of Limonene, Linalool and α-Terpineol Combinations in Pinot Gris Wine
Source: Foods. 2023 Jun 16;12(12):2389. doi: 10.3390/foods12122389 (PMC10297329; doi:10.3390/foods12122389)
Supplement: Supplementary file 1 [file foods-12-02389-s001.zip › foods-2259465-supplementary.pdf]

Table S1. Final concentration and CAS# of aroma compounds used in the wine base*

Compounds	CAS#	µg/L
Ethanal	75-07-0	5000.00
2,3-Butanedione	431-03-8	50.00
Hexan-1-ol	111-27-3	70.00
Methionol	505-10-2	31.00
Butanoic acid	107-92-6	650.00
Decanoic acid	334-48-5	200.00
2-methylpropanoic acid	79-31-2	800.00
2-methylbutanoic acid	116-53-0	100.00
3-Methylbutanoic acid	503-74-2	700.00
2-methylpropyl ethanoate	123-92-2	15.00
Ethyl phenylacetate	101-97-3	400.00
Ethyl 3-methylbutanoate	108-64-5	15.00
ethyl 2-methylpropanoate	97-62-1	5.00
Ethyl 2-methylbutyrate	7452-79-1	10.00
Ethyl Butanoate	105-54-4	120.00
Ethyl Decanoate	110-38-3	200.00
Ethyl Hexanoate	123-66-0	350.00
Ethyl Octanoate	106-32-1	900.00
Hexanoic Acid	142-62-1	1000.00
Octanoic Acid	124-07-2	4000.00
2-methylpropan-1-ol	78-83-1	35000.00
Ethyl Acetate	141-78-6	30000.00
3-Methylbutyl ethanoate	123-92-2	2000.00
Acetic Acid	64-19-7	100000.00

* The concentration was made with average of concentrations found in wines analyzed by Song et al., 2018

Table S2. Concentration (μg/L) of terpenes used for CATA1 sensory panel.

Wines	(S)-(−)-Limonene	(R)-(+)-Limonene	Linalool	α-terpineol
Model 18				
Model a	1.1	0.9		
Model b	1.1	0.9	17	
Model c	1.1	0.9		20
Model d	1.1	0.9	17	20
Model e	2.2	1.8		
Model f	2.2	1.8	13	
Model g	2.2	1.8		15
Model h	2.2	1.8	13	15
Model i	2.2	1.8	25	
Model j	2.2	1.8		32
Model k	2.2	1.8	25	32
Model l			17	
Model m				20

Model a to k had a 55:45 ratio of Limonene

Table S3. Multiple pairwise comparisons using the critical difference (Sheskin) procedure

Sample	Relative frequency	Groups						
Lavender	0.040	A						
Ginger	0.053	A						
Grapefruit	0.063	A	B					
Asparagus	0.063	A	B					
Lime	0.069	A	B					
Mango	0.082	A	B	C				
Guava	0.082	A	B	C				
Passionfruit	0.090	A	B	C				
Pineapple	0.095	A	B	C				
Lemon	0.101	A	B	C				
Rose	0.108	A	B	C				
Melon	0.111	A	B	C				
Grass	0.111	A	B	C				
Allspice	0.124	A	B	C	D			
Orange	0.130	A	B	C	D			
Lychee	0.151		B	C	D			
Nutty	0.156		B	C	D			
Jasmine	0.164			C	D			
Lilac	0.172			C	D	E		
Stone fruit	0.206				D	E	F	
Honeysuckle	0.214				D	E	F	
Pome fruit	0.259					E	F	
Dried fruit	0.286						F	G
Honey	0.362							G
